# Deletion of a Conserved *cis*-Element in the *Ifng* Locus Highlights the Role of Acute Histone Acetylation in Modulating Inducible Gene Transcription

**DOI:** 10.1371/journal.pgen.1003969

**Published:** 2014-01-09

**Authors:** Anand Balasubramani, Colleen J. Winstead, Henrietta Turner, Karen M. Janowski, Stacey N. Harbour, Yoichiro Shibata, Gregory E. Crawford, Robin D. Hatton, Casey T. Weaver

**Affiliations:** 1 Department of Pathology, University of Alabama at Birmingham, Birmingham, Alabama, United States of America; 2 Department of Microbiology, University of Alabama at Birmingham, Birmingham, Alabama, United States of America; 3 Institute for Genome Sciences & Policy, Duke University, Durham, North Carolina, United States of America; Washington University, United States of America

## Abstract

Differentiation-dependent regulation of the *Ifng* cytokine gene locus in T helper (Th) cells has emerged as an excellent model for functional study of distal elements that control lineage-specific gene expression. We previously identified a *cis*-regulatory element located 22 kb upstream of the *Ifng* gene (Conserved Non-coding Sequence -22, or CNS-22) that is a site for recruitment of the transcription factors T-bet, Runx3, NF-κB and STAT4, which act to regulate transcription of the *Ifng* gene in Th1 cells. Here, we report the generation of mice with a conditional deletion of CNS-22 that has enabled us to define the epigenetic and functional consequences of its absence. Deletion of CNS-22 led to a defect in induction of *Ifng* by the cytokines IL-12 and IL-18, with a more modest effect on induction via T-cell receptor activation. To better understand how CNS-22 and other *Ifng* CNSs regulated *Ifng* transcription in response to these distinct stimuli, we examined activation-dependent changes in epigenetic modifications across the extended *Ifng* locus in CNS-22-deficient T cells. We demonstrate that in response to both cytokine and TCR driven activation signals, CNS-22 and other *Ifng* CNSs recruit increased activity of histone acetyl transferases (HATs) that transiently enhance levels of histones H3 and H4 acetylation across the extended *Ifng* locus. We also demonstrate that activation-responsive increases in histone acetylation levels are directly linked to the ability of *Ifng* CNSs to acutely enhance Pol II recruitment to the *Ifng* promoter. Finally, we show that impairment in IL-12+IL-18 dependent induction of *Ifng* stems from the importance of CNS-22 in coordinating locus-wide levels of histone acetylation in response to these cytokines. These findings identify a role for acute histone acetylation in the enhancer function of distal conserved *cis*-elements that regulate of *Ifng* gene expression.

## Author Summary

Differentiation of multipotent naïve T cell precursors into functionally mature effector cells that control different types of immune responses is an excellent model to study lineage-specific regulation of gene expression. A number of *cis*-regulatory elements have been reported to control expression of the gene that encodes the cytokine IFN-γ which is a signature product of effector T cells of the Th1 lineage. However, none of these elements has been analyzed for effects on gene expression and chromatin remodeling through deletional analysis in the native *Ifng* gene locus. Here we have generated mice in which a key element previously implicated in control of *Ifng* gene expression (CNS-22) was conditionally deleted from the genome. Th1 cells in which CNS-22 was deleted had activation-specific deficits in *Ifng* expression and demonstrated defects in epigenetic changes across the *Ifng* locus. Mapping epigenetic consequences of CNS-22 deletion led to identification of acute hyperacetylation of histones immediately flanking this and other *cis*-regulatory elements that was associated with *Ifng* gene transcription, as well as more global defects in histone acetylation. These findings support a mechanism whereby regulatory sites that have acquired baseline histone acetylation marks during lineage specification undergo acute, activation-dependent increases in histone acetyl transferase activity that enhance transcription of inducible genes.

## Introduction

Distal regulatory elements including locus control regions, enhancers, silencers and boundary elements play important roles in regulating cell lineage-specific activation and repression of genes [1], [2], [3], [4], [5], [6]. In addition to genome-wide studies to document and classify putative distal regulatory sites, studies on individual gene loci have been instrumental in shaping our understanding of *cis* element function [7], [8], [9]. Although genes expressed in several cell types including embryonic stem cells (*Hox* genes), B-lineage cells (immunoglobulin genes) and erythroid cells (globin genes) have emerged as important models to understand eukaryotic transcription, cytokine genes expressed in T-helper cells are particularly attractive models to study lineage specific regulation. Primary human and murine naïve Th cells can be readily isolated in large numbers and be differentiated into functionally and transcriptionally distinct Th cells as exemplified by Th1, Th2, Th17, and T-regulatory (Treg) cell subsets [10], [11], [12]. In particular, genes that encode Th2 cytokines, comprised of the *Il4*, *Il13* and *Il5* genes and the *Ifng* gene transcribed in Th1 cells have emerged as key models to the study lineage-appropriate gene expression [8], [12] [13], [14].

The importance of distal elements in regulating expression of human and mouse genes that encode IFN-γ was first recognized in mice transgenic for a bacterial artificial chromosome (BAC) that encompassed ∼190 kb flanking the human *IFNG* gene, which, unlike transgenes that contained more limited flanking sequence, conferred lineage-specific expression of human IFN-γ in mouse Th1 cells [15], [16]. Subsequently, we reported a murine *Ifng-Thy1.1* BAC reporter transgene that spanned ∼160 kb surrounding *Ifng*, which also demonstrated lineage- and activation-specific expression [17], [18], suggesting that distal elements required for lineage specific expression of *Ifng* were contained in this region. Based on recruitment of CTCF and Rad21 (a cohesin), the *IFNG* and *Ifng* loci are predicted to extend from −63 to +119 kb [19] and −70 kb to +66 kb [20], respectively. Within these boundary elements, at least nine conserved non-coding sequences (CNS) have been identified based on the high degree of sequence conservation at these sites in multiple mammalian species [2], [3].

Using ChIP-qPCR and promoter-reporter assays, a subset of these CNSs was probed for *trans*-factor binding and histone modifications in early studies [18], [21], [22], [23], [24]. More recently, DNase-chip [25] and DNase-seq [20] have been employed to map chromatin conformation of the extended *Ifng* locus in multiple T cell lineages. In parallel, analyses of *trans* factor recruitment to these *cis* elements have facilitated their further functional mapping. T-bet [18], [22], [23], STAT4 [26], [27], Runx3 [28] and members of the NF-κB [29] and NFAT [23] families of transcription factors have been demonstrated to interact with *cis* elements across the *Ifng* locus [13].

To date, the functions of four *Ifng/IFNG* CNSs have been examined by deletional analyses in the context of *Ifng* or *IFNG* BAC transgenes [18], [30]. Deletion of the human homolog of *Ifng* CNS-34 (*IFNG* CNS-30) from a 190 kb human *IFNG* transgene resulted in impaired expression of *IFNG* in T cells but not NK cells [30] suggesting that one or more of these regulatory sequences may have lineage-specific functions. In our own studies, deletion of CNS-22 from the *Ifng-Thy1.1* reporter transgene led to nearly complete ablation of Thy1.1 reporter expression in both T cells and NK cells [18]. In more recent studies using *IFNG* BAC transgenes, human homologs of CNSs −6 and +17–19 were also shown to be important enhancers of *IFNG* transcription [31]. CNS-22 is of particular interest, since it is hypersensitive not only in Th1 cells, but also in naïve precursors, suggesting that CNS-22 might act as a principal site for recruitment of “pioneer” *trans* factors that initiate remodeling of the *Ifng* locus [25]. Further, CNS-22 remains hypersensitive to DNase I in Th2 and Th17 cells despite repression of *Ifng* expression in these lineages, suggesting that CNS-22 may be involved in *Ifng* transcriptional silencing in addition to its role in transcriptional activation [25], [29]. Taken together with the profound effect of selective CNS-22 deletion on BAC reporter expression, these findings led us to speculate that this element could be an important node for directing chromatin remodeling of the *Ifng* locus [18].

Therefore, we generated mice targeted for conditional deletion of CNS-22 in the endogenous *Ifng* loci (*Ifng.CNS-22^fl/fl^*) to enable mapping of epigenetic modifications not previously possible using BAC transgenic mice. Here, we report the functional consequences of the deletion of CNS-22 on epigenetic remodeling and gene expression of the *Ifng* locus in naïve and differentiated T cells. We find that deletion of CNS-22 in Th1 cells results in greater impairment of *Ifng* transcription in response to IL-12 plus IL-18 than that induced by TCR dependent signaling. This is associated with a defect in the deposition of histone acetylation marks on nucleosomes immediately flanking CNS-22 as well as those distributed distally across the *Ifng* locus. These findings identify a previously unappreciated role for activation-induced modulation of HAT activity in driving cytokine gene transcription.

## Results

### Functional characterization of *Ifng.CNS-22* ^−/−^ mice

**Figure 1:**
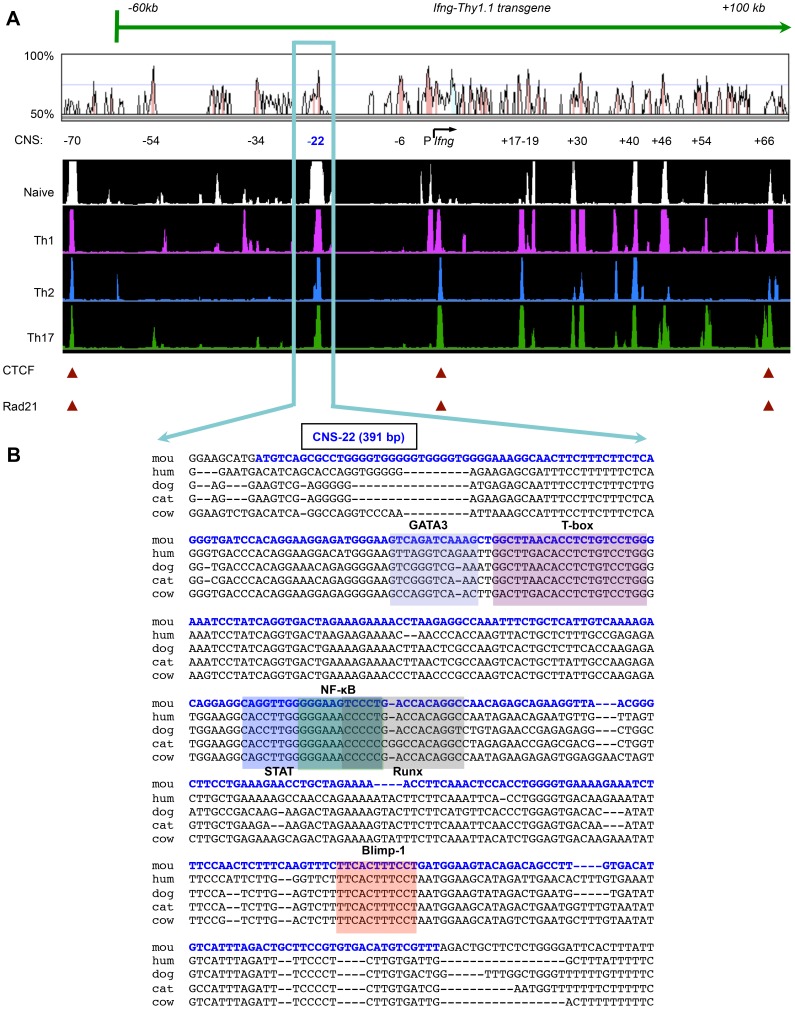
*Ifng*-CNS-22: An evolutionarily conserved distal regulatory hub. (**A**) Syntenic regions of human and murine *Ifng* gene loci are shown aligned using the VISTA browser. CNSs represent regions of at least 100 bp in length that exhibit at least 70% homology between these two species. Locations of key *Ifng* CNSs including CNS-22 are listed below. Aligned below are DNase I hypersensitivity tracks of the extended *Ifng* locus generated from naïve CD4^+^, Th1, Th2 and Th17 cells. DNase I tracks represent averages from two independent runs. CTCF and cohesion binding sites that represent putative boundaries of the *Ifng* locus are highlighted below. Also shown is the extent of the *Ifng-Thy1.1* (green) BAC transgene that we previously employed to study *cis* regulation of IFN-γ. This transgene spanned from −60 kb upstream to +100 kb downstream of *Ifng*, but excluded a more recently identified insulator sequence located 70 kb upstream of *Ifng*. (**B**) Genomic sequence of region deleted to generate CNS-22^−/−^ mice is shown aligned against syntenic sequences from the genomes of multiple mammalian species. The murine CNS-22 sequence deleted to generate CNS-22^−/−^ mice is highlighted in blue. The same sequence highlighted in this figure was also deleted from the *Ifng-Thy1.1* transgene in our previous studies. Also highlighted are *trans* factor binding sites of transcription factors that have been demonstrated to bind to CNS-22.

*Ifng.CNS-22^fl/fl^* mice were generated using a targeting construct in which 391 bp corresponding to CNS-22 in the *Ifng* locus was flanked by *loxp* sites to enable *Cre*-mediated excision (**Fig. S1A, B**) [18], [32], [33]. CNS-22 resides in a broad DNase I hypersensitive (HS) site that encompasses CNS-22 at the 3′ end (**Fig. S1C**). Cre-mediated excision of this element deletes several evolutionarily conserved *trans*-factor binding sequences, including sites that recruit T-bet, STAT4, RelA and Runx3 (**Fig. 1B**) [18], [28], [29]. *Ifng.CNS-22* was deleted in the germline by crosses with EIIa.Cre mice, such that all cells, including T cells, were CNS-22–deficient (henceforth referred to as CNS-22^−/−^ mice). Phenotypically, CNS-22^−/−^ mice were indistinguishable from littermate controls. Numbers of CD4^+^ and CD8^+^ T cells in the periphery were comparable to wildtype controls (unpublished observations).

**Figure 2:**
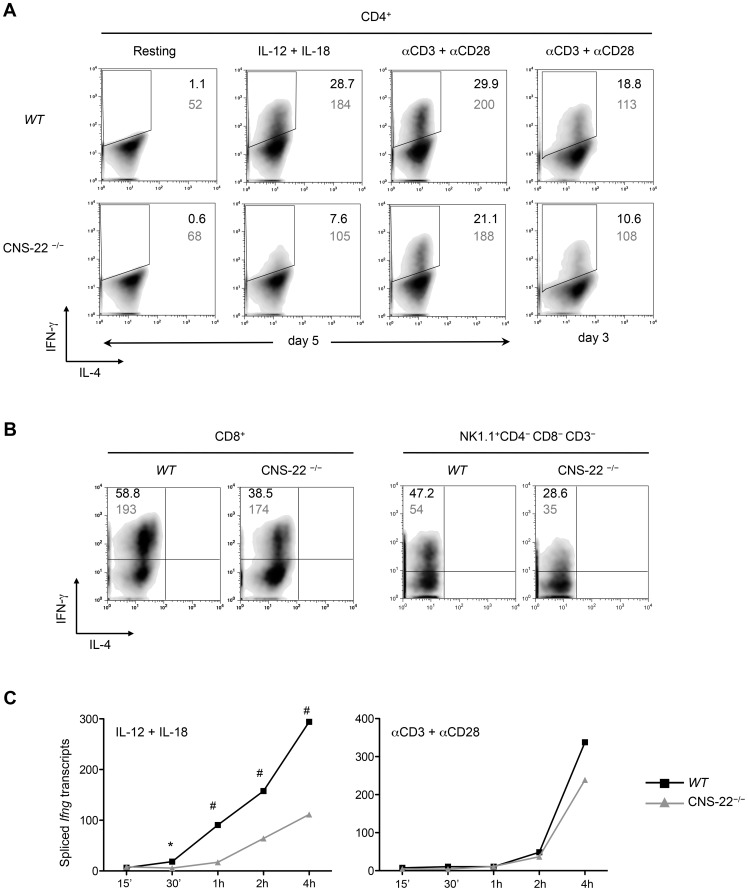
IL-12+IL-18 driven *Ifng* transcription is compromised in CNS-22^−/−^ T cells and NK cells. (**A**) CD4^+^ T cells derived from OT-II transgenic WT and CNS-22^−/−^ mice were differentiated with 2 ng/ml IL-12, 5 µg/ml ova-peptide (ISQAVHAAHAEINEAGR) and cultured with CD4-depleted irradiated feeder cells derived from *Il12a* ^−/−^ mice. Cells were reactivated for 4 h as described in methods and expression of IFN-γ was assessed by flow cytometric analysis following intracellular staining. Percentages of viable, IFN-γ^+^ T cells are indicated by black numbers and mean fluorescence intensities (MFI) of IFN-γ^+^ cells are indicated in grey. Data are representative of at least three independent experiments. (**B**) CD8^+^ T cells isolated from WT and CNS-22^−/−^ mice were differentiated with 2.5 µg/ml of anti-CD3 antibody, 2 ng/ml IL-12 for 3 days and reactivated for 4 h with IL-12+IL-18 and subject to intracellular staining. For evaluating IFN-γ expression in NK cells, following depletion of both CD4^+^ and CD8^+^ T cells bulk splenocytes were activated with IL-12+IL-18 for 4 h and subject to intracellular staining. Percentages of viable, IFN-γ^+^ T/NK cells are indicated in black and MFI of IFN-γ^+^ cells are indicated in grey. Data are representative of at least three independent experiments. (**C**) Total RNA from anti-CD3+anti-CD28 or IL-12+IL-18 stimulated WT and CNS-22^−/−^ Th1 cells was isolated at indicated time points and reverse-transcribed to generate cDNA. Transcript levels were measured by RT-PCR and relative levels of spliced transcripts were calculated by normalization against levels of spliced transcripts in resting Th1 cells. Data represent means from at least three independent experiments. Statistical analyses were carried out on means and standard errors from three independent experiments * p<0.01, # p<0.05, stimulated WT versus CNS-22^−/−^.

To examine the impact of CNS-22 deletion on *Ifng* gene expression, naïve CD4^+^ T cells from OT-II transgenic WT and CNS-22^−/−^ mice were differentiated *ex vivo* in the presence of a low or high concentration of IL-12 and the expression of IFN-γ induced by restimulation with IL-12+IL-18 or TCR signaling was examined (**Fig. S2A**). Irrespective of the concentration of IL-12 used, Th1 cells generated from CNS-22^−/−^ mice were significantly impaired in their expression of IFN-γ in response to IL-12+IL-18 restimulation, whereas a deficit in IFN-γ expression in response to TCR stimulation was only apparent for cells differentiated with the low concentration of IL-12 **(** **Figs. 2A** **and S2A**). In contrast to cells restimulated with IL-12+IL-18, which showed a similar deficit in IFN-γ expression whether activated on day 3 or day 5 of differentiation, the impact of CNS-22 deletion on impaired TCR-driven induction of IFN-γ was more pronounced in Th1 cells restimulated on day 3 (**Figs. 2A** **and S2A**, and data not shown). The impairment of *Ifng* transcription was not due to alterations in the expression of key *trans* factors, as there were no significant differences in expression of *Tbx21* or *Runx3* in CNS-22-deficient T cells (**Fig. S2B**). IL-12+IL-18 driven induction of *Ifng* was also considerably impaired in Tc1 cells and in NK cells from in CNS-22^−/−^ mice (**Fig. 2B**). Impairment of *Ifng* expression was also observed in vivo for CNS-22-deficient T cells responding to infection with *Listeria monocytogenes*, an intracellular bacterial pathogen that induces a type 1 immune response (**Figs. S3A–C**).

By comparing the kinetics of induction of *Ifng* transcripts in Th1 cells, we found that early transcription of *Ifng* in response to IL-12+IL-18 was significantly compromised in the absence of CNS-22, while there was no significant impairment in response to TCR signaling (**Fig. 2C**), reinforcing the observation that induction of transcription via the TCR signaling pathway was less dependent on CNS-22 function. Finally, Th1 cells derived from CNS-22^+/−^ mice showed an intermediate, copy number–dependent impairment in IL-12+IL-18 driven *Ifng* gene transcription (unpublished observations), demonstrating that CNS-22 plays an obligatory role in integrating activating signals downstream of IL-12 and IL-18 receptors [29]. Taken together with our previous findings that CNS-22 recruits STAT4 and NF-κB downstream of the IL-12 and IL-18 receptors, respectively [13], whereas TCR signaling does not activate STAT4, these results suggest that CNS-22 is more important as a STAT4-dependent enhancer for acute activation of *Ifng* transcription, although they do not exclude a critical contribution for CNS-22 as node for epigenetic modifications of the *Ifng* locus during the development of immune cells.

### CNS-22 plays a developmental stage-specific role in remodeling of the *Ifng* locus

**Figure 3:**
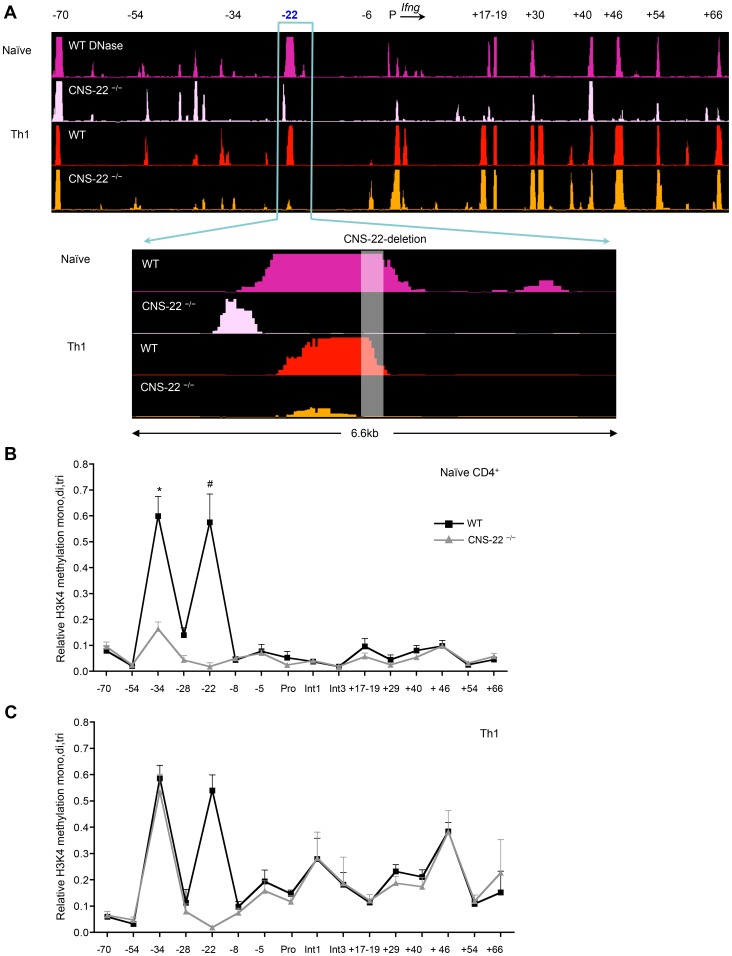
CNS-22 plays a limited role in long-range remodeling of the extended *Ifng* locus. (**A**) Naïve CD4^+^ T cells and Th1 cells derived from WT and CNS-22^−/−^ mice were subject to DNase-chip as previously described [25] DNase I HS sites across the *Ifng* locus were analyzed with ACME using peak-calling thresholds set to a confidence limit of 95% [47] and visualized with the IGB browser [48]. DNaseI sites are shown aligned against a VISTA plot that highlights locations of *Ifng* CNSs. Data are representative of at least two independent experiments. (**B**, **C**) Naïve CD4^+^ T cells and Th1 cells derived from WT and CNS-22^−/−^ mice were analyzed by ChIP using an antibody that recognizes all three methylation states of H3K4. Relative H3K4 methylation levels were calculated by comparisons with no antibody controls and are represented as a fraction of the H3K4 methylation observed at 16S ribosomal protein (16Srp) promoter, which was assigned a value of 1. Data represent mean±SEM from at least three independent experiments. * p<0.01, # p<0.05, stimulated WT versus CNS-22^−/−^.

Our previous studies demonstrated that deletion of CNS-22 in the context of the *Ifng-Thy1.1* reporter transgenic locus resulted in nearly complete ablation of Thy1.1 reporter expression in Th1, Tc1 and NK cells [13]. This led us to hypothesize that CNS-22 was essential for orchestrating remodeling of the *Ifng* locus in this transgene in addition to its role as a key enhancer (ref. [18], and see **Fig. 1A**). While we confirm here the importance of CNS-22 as a positive modulator of *Ifng* transcription (**Fig. 2**), the less pronounced impairment of *Ifng* transcription found in CNS-22^−/−^ Th1 cells suggested that deletion of CNS-22 from endogenous *Ifng* loci might not have as fundamental a role in regulating chromatin accessibility during Th1 cell development as speculated (see below). Nevertheless, IL-12 dependent acquisition of *Ifng* competence was considerably delayed in CNS-22^−/−^ CD4^+^ T cells (**Fig. 2A**). Since we had previously documented that CNS-22 was permissive in naïve CD4^+^ T cells (ref. [13], and see also **Fig. 3A**), we hypothesized that CNS-22 might play an obligatory role in epigenetic remodeling the *Ifng* locus prior to Th1 differentiation.

To examine this, we performed DNase-chip and ChIP-qPCR-based analysis of histone 3 lysine 4 methylation (H3K4) to assess the epigenetic status of the extended *Ifng* locus in CNS-22-deficient naïve CD4^+^ T cells and Th1 cells (**Fig. 3**). In agreement with our previous study [25], we found that several key distal elements in the *Ifng* locus are hypersensitive (HS) to DNase I in naïve WT CD4^+^ T cells (**Fig. 3A**). Notably, naïve cells from CNS-22^−/−^ mice showed marked reduction or a lack of hypersensitivity at most sites identified in WT cells, including those at CNSs +17–19, +30, +46, +54 and +66 (**Fig. 3A**). In contrast, DNase I HS sites at the upstream CTCF binding insulator element (−70) and CNS+40 arise in a CNS-22–independent fashion, indicating that even prior to exposure to lineage-specifying signals, multiple nodes appear poised to initiate reorganization of the extended *Ifng* locus. We also observed a prominent HS peak immediately upstream of CNS-22 in naïve CNS-22^−/−^ CD4^+^ T cells (**Fig. 3A**), although this was much less pronounced following Th1 differentiation. While the basis for this is unclear, it is plausible that some functions of CNS-22 are not compromised by deletion of the core regulatory element.

In accord with the functional analyses of *Ifng* expression in CNS-22^−/−^ Th1 cells, defects in locus-wide remodeling apparent in naïve CNS-22–deficient CD4^+^ T cells were largely reversed upon Th1 differentiation (**Fig. 3A**). Specifically, the pattern of DNAse HS at regulatory elements downstream of the *Ifng* gene was nearly indistinguishable from WT Th1 cells, indicating that differentiation-dependent remodeling of the *Ifng* locus is largely independent of CNS-22.

**Figure 4:**
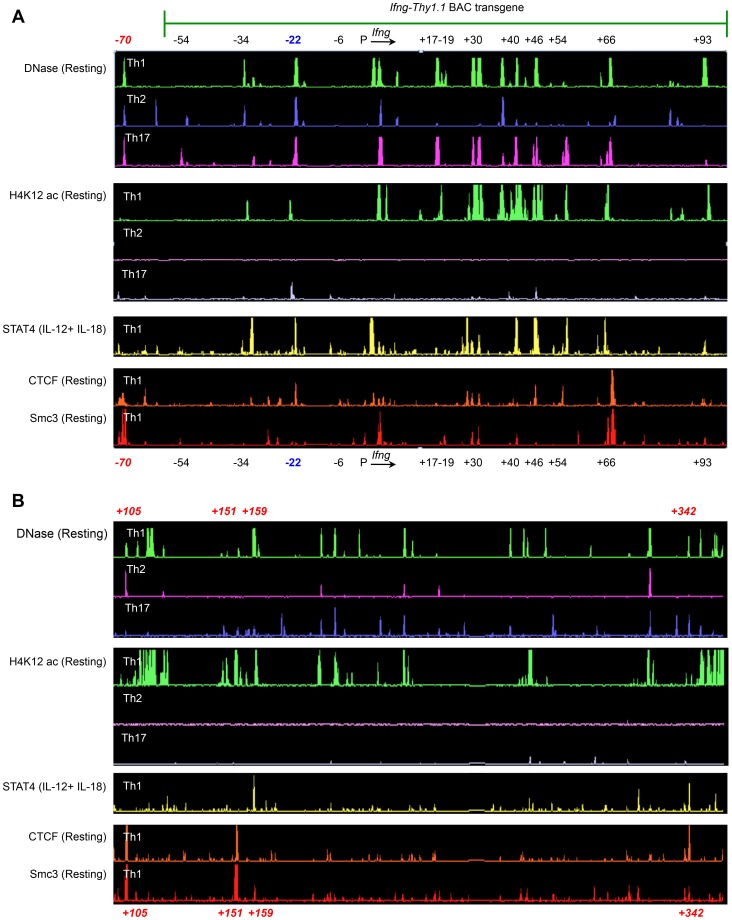
Identification of putative regulatory elements greater than 100*Ifng* gene. (**A–B**) Naïve CD4^+^ T cells from OT-II transgenic WT mice were differentiated under Th1, Th2 or Th17 polarizing conditions. Cells were left unstimulated or activated with IL-12+IL-18 for 1.5 h and subject to ChIP-chip. To identify STAT4, Smc3 and CTCF binding sites and to define H4K12ac-enriched regions across the *Ifng* locus, peak calling was carried out using a previously described algorithm for capturing microarray enrichment (ACME) [49]. Peak-calling thresholds were set to a confidence limit of 95% and visualized with the IGB browser [48]. Data are representative of at least two independent experiments.

To complement DNase-chip analyses, we also carried out ChIP-qPCR to evaluate H3K4 mono-, di- and tri methylation status is Th1 cells derived from either WT or CNS-22^−/−^ mice (**Fig. 3B**). In parallel to the DNase I HS results, deposition of permissive H3K4 methylation marks in naïve CD4^+^ T cells was significantly impaired in the absence of CNS-22, particularly at CNS-34 and non-conserved site −28 (**Fig. 3B**). However, levels of H3K4 methylation was comparable at both these sites in CNS-22–deficient and WT Th1 cells (**Fig. 3C**), indicating that CNS-22 was dispensable for differentiation-dependent remodeling of the *Ifng* locus in Th1 cells. Thus, the effect of CNS-22 deletion in the context of the endogenous *Ifng* was less pronounced than in the context of an *Ifng* BAC transgenic locus we reported previously (**Figs. 1A** and **4A**).

We hypothesized that the observed differences in the contribution of CNS-22 to *Ifng* transcription from the endogenous or transgenic loci might be attributed to the absence of key distal regulatory sequences in the BAC-transgene. The BAC-transgenic reporter included 60 kb upstream and 100 kb downstream of the *Ifng* gene and consequently lacked a more recently defined insulator/chromatin looping sequence that is located 70 kb upstream of *Ifng* (**Fig. 4A**) [19], [20]. In addition, it was also possible that some distal elements that regulate *Ifng* lie greater than 100 kb downstream of *Ifng*. The first clue that this was likely came from ChIP-chip analyses carried out in our previous studies to identify STAT4 and RelA binding enhancer sequences [29]. We discovered at least one prominent STAT4 binding site that was 159 kb downstream of the *Ifng* gene (**Fig. 4B**). This site was hypersensitive to DNase I in Th1 cells, but not in Th2 or Th17 cells suggesting that it functioned as a STAT4 responsive module in Th1 cells.

To further explore the possibility that potential regulatory elements may reside greater than 100 kb downstream of *Ifng* and therefore would be excluded from the transgene, we carried out ChIP-chip analyses to map recruitment of CTCF and a cohesion family member Smc3. We identified at least three prominent CTCF and Smc3 binding sites +105, +151 and +342 kb downstream of *Ifng* (**Fig. 4B**). At all three sites, we observed Th1-specific acquisition of histone H4 Lysine-12 acetylation (H4K12ac) marks indicating that these elements are functional in Th1 cells (**Fig. 4B**). Therefore, we speculate that exclusion of these downstream regulatory sequences and the CTCF binding site located −70 kb upstream of *Ifng* in our previous BAC-transgenic studies may have magnified the role of CNS-22 in coordinating *Ifng* expression. Nonetheless, by deleting CNS-22 at the endogenous locus, we demonstrate here that CNS-22 is an obligate enhancer that is primarily important in regulating IL-12+IL-18 dependent induction of *Ifng* in Th1, Tc1 and NK cells.

### CNS-22 modulates both proximal and distal increases in activation-driven histone acetylation

Th1/Tc1 specific transcriptional competence of the *Ifng* gene is marked not only by acquisition of H3K4 mono, di and tri- methylation marks, but also acetylation of several lysine residues on histones H3 and H4 across the extended *Ifng* locus [18]. Acetylation of histone residues H4K16, H3K4 and H4K12 is present not only at promoters, but also throughout the extended loci of transcribed genes [34]. More recently, acetylation of H3K27 has been correlated with lineage-specific activity of enhancers [35]. We initially examined these modifications and others (unpublished observations), and chose to focus on H4K12 acetylation due to the greater efficiency, specificity and dynamic range of chromatin immunoprecipitation observed for this particular histone modification. Acquisition of H4K12ac marks at CNS-22 and globally across the extended *Ifng* locus was specific to differentiated Th1 cells (**Fig. 4**) [18], [26]. Similarly, acquisition of lineage-specific H4K12ac marks correlated with transcriptional competence at other T lineage-specific gene loci, including the *Il17a/Il17f* and *Il4-Il13-Il5* gene clusters in differentiated Th17 and Th2 cells, respectively (**Fig. S4**).

In a previous study we noted that H4 acetylation at multiple distal sites in the *Ifng* locus, including CNS-22, was increased by signals that led to acute induction of *Ifng* transcription in both Th1 and Tc1 cells [18]. Notably, the activation dependent change in H4 acetylation at these non-conserved sites was much greater than changes observed at *Ifng* CNSs [18]. We therefore speculated that inducible acquisition of histone acetylation marks at these distal non-conserved sites might be linked to activation-induced transcription of *Ifng*, and that CNS-22 and other CNSs in the *Ifng* locus might act as nucleation sites for enhanced recruitment or activation of HATs that decorate the histones of neighboring nucleosomes. If true, induction of *Ifng* transcription in Th1 and Tc1 cells would be predicted to correlate with acute increases in histone acetylation at sites immediately flanking CNSs across the *Ifng* locus. Accordingly, deletion of CNS-22 would be predicted to impair acquisition of these inducible acetylation marks.

**Figure 5:**
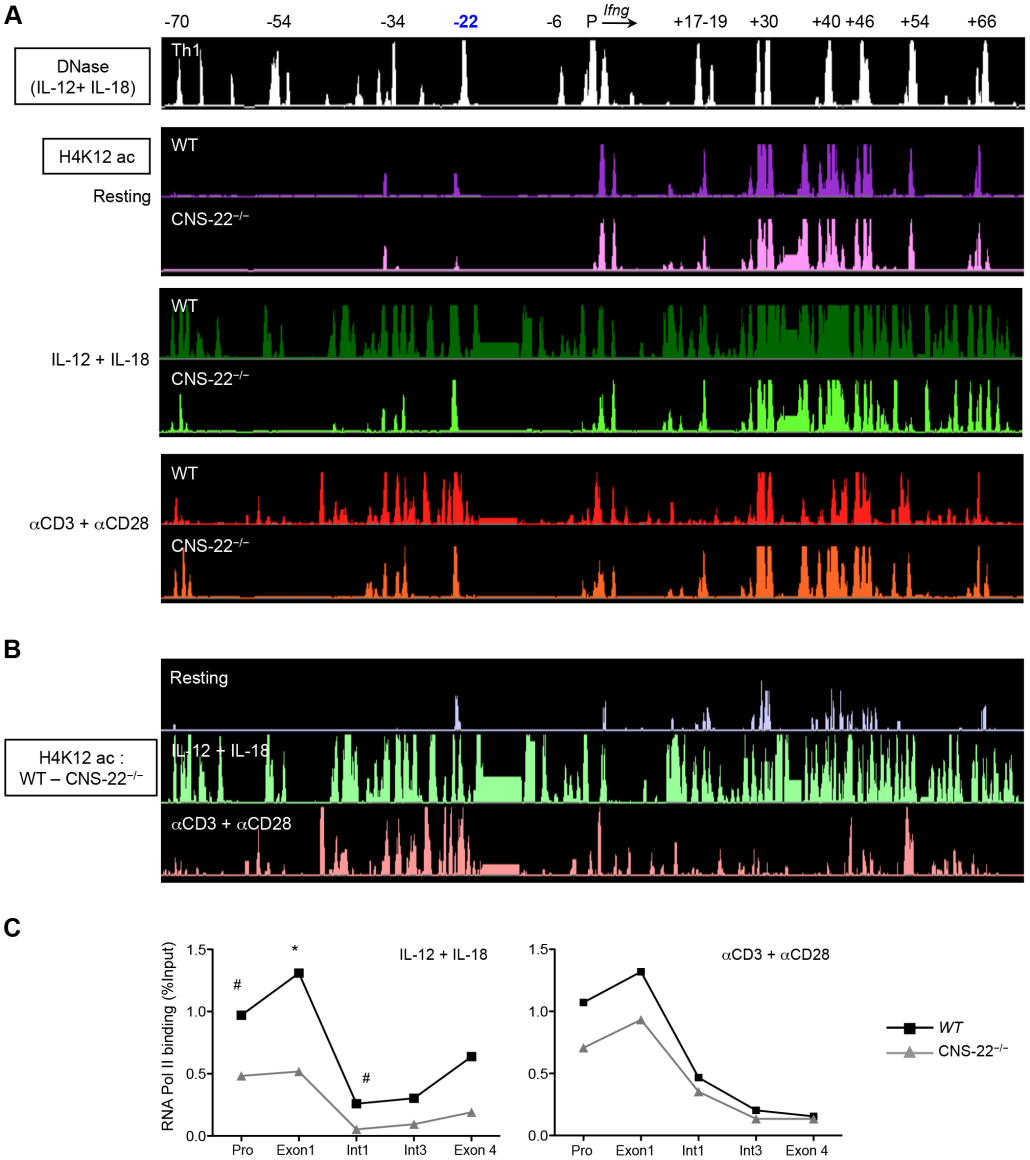
Deletion of CNS-22 leads to prominent defects in IL-12+IL-18 dependent modulation of histone hyperacetylation. (**A**) Th1 cells generated from either WT or CNS-22^−/−^ mice were either left unstimulated or activated with IL-12+IL-18 for 1.5 h or anti-CD3 and anti-CD28 for 3 h and then subject to ChIP-chip with antibodies against H4K12ac. Levels of H4K12ac were analyzed as described in Fig. 3 and visualized using the IGB browser. (**B**) H4K12ac levels across the *Ifng* locus documented in WT Th1 cells were normalized against H4K12ac levels documented in CNS-22^−/−^ Th1 cells to visualize in a semi-quantitative manner the magnitude to which locus-wide acquisition of H4K12ac marks was impaired in the absence of CNS-22. (**C**) Th1 cells generated from WT or CNS-22^−/−^ mice were restimulated with anti-CD3+anti-CD28 for 3 h or IL-12+IL-18 for 1.5 h and recruitment of RNA-Pol II to the *Ifng* gene was assessed by ChIP-qPCR. RNA-Pol II recruitment is shown as a percentage of input DNA. Data represent means from at least three independent experiments. Statistical analyses were carried out on means and standard errors from three independent experiments * p<0.01, # p<0.05, stimulated WT versus CNS-22^−/−^.

To test this, we evaluated levels of histone H4 acetylation in the immediate vicinity of CNS-22 in resting and activated Th1 cells. In response to both IL-12+IL-18 and TCR driven activation signals, a prominent increase in levels of H4 acetylation at sites within ∼1 kb flanking CNS-22 (−22.9, −22.4, −21.7) was observed (**Fig. S5**). We also observed similar activation-induced acetylation at *Ifng* CNSs −34 and +46 (see **Fig. 5**, below, and data not shown). We subsequently performed ChIP-qPCR experiments in Th1 cells derived from CNS-22^−/−^ mice to examine whether activation-driven acquisition of acetylation marks at −22.9, −22.4 and −21.7 was dependent on CNS-22. While resting levels of H4 acetylation at −22.9, −22.4 and −21.7 were unaffected by deletion of CNS-22, acute hyperacetylation at these sites in response to both IL-12+IL-18 and TCR signaling was significantly impaired (**Figs. S5B, C**). These results led us to speculate that the ability of CNS-22 to acutely recruit HATs in response to external stimuli is linked to its ability to enhance *Ifng* transcription. This also led us to ask two further questions. Firstly, is activation-driven acquisition of H4 acetylation marks unique to CNS-22, or is this a common mechanism employed by multiple *Ifng* enhancers? Secondly, since Th1 cells generated from CNS-22^−/−^ mice show a more prominent defect in IL-12+IL-18 driven activation signals, but acquisition of H4 acetylation near CNS-22 was significantly impaired in response to both IL-12+IL-18 and TCR reactivation signals, does CNS-22 regulate histone acetylation only within its immediate vicinity or does it influence histone acetylation levels at more distal sites as well? To address these two questions, we have carried out ChIP-chip experiments to examine H4K12 acetylation in resting and activated Th1 cells generated from WT and CNS-22^−/−^ mice.

In resting Th1 cells, H4K12ac marks were confined to the regions in immediate proximity to the CNSs themselves and deletion of CNS-22 did not significantly alter acquisition of these marks (**Fig. 5A**). Upon activation with either IL-12+IL-18 or with anti-CD3+anti-CD28, there was robust, extensive deposition of acute acetylation marks that extended beyond the CNSs to more distal, non-conserved sequences (**Fig. 5A**). As predicted by conventional ChIP-qPCR, acquisition of acetylation marks in the immediate vicinity of CNS-22 was absolutely dependent upon CNS-22 irrespective of the mode of activation (**Fig. S5**). However, normalization and comparison on H4K12ac levels between WT and CNS-22^−/−^ Th1 cells revealed a much more prominent defect in IL-12+IL-18 dependent acquisition of H4K12ac marks extending across the *Ifng* locus (**Fig. 5B**). Specifically, in response to TCR driven acquisition of H4K12ac marks, deletion of CNS-22 impaired acquisition of H4K12ac in proximity to CNS-22 as well as in enhancers upstream of CNS-22 (CNSs −54 and −34), but had little to no effect on deposition of acetylation marks at CNSs downstream of the *Ifng* gene (**Fig. 5B**). In contrast, in CNS-22–deficient Th1 cells IL-12+IL-18 dependent acquisition of H4K12ac was globally altered across the *Ifng* locus such that defects in acquisition of this epigenetic mark were evident at multiple CNSs downstream of the *Ifng* gene (**Fig. 5B**). Therefore, the greater defect in acute acquisition of H4K12ac marks in response to IL-12+IL-18 might account for the greater impairment of IL-12+IL-18 dependent induction of *Ifng* compared to that observed following TCR driven induction.

### Acute, activation-dependent histone acetylation regulates inducible recruitment of RNA Pol II to the *Ifng* gene

The positive correlation between accumulation of histone acetylation marks that accompany lineage specification and induction of gene transcription is well established. More recently, the recruitment of bromodomain-containing HATs, particularly p300 and CBP, to distal regulatory sequences has been used to identify lineage-specific enhancers [36], [37]. In view of the role played by CNS-22 in the HAT-mediated modification of the *Ifng* locus, we interrogated its association with p300 (**Fig. S6**). Binding of p300 to CNS-22 and other CNSs across the *Ifng* locus was evident in resting and activated Th1 cells, and with few exceptions the level of p300 binding — whether to CNS-22 or other CNSs across the locus — was not substantially altered by activation. This finding, coupled with the marked impairment in acquisition of H4K12 acetylation in CNS-22–deficient Th1 cells following IL-12+IL-18 stimulation, led us to speculate that activation-induced enhancement of HAT activity at CNS-22 resulted in increased recruitment of RNA Pol II to the *Ifng* promoter. To examine this, we compared recruitment of RNA Pol II to the *Ifng* gene in resting Th1 cells and Th1 cells activated by IL-12+IL-18 or TCR stimulation (**Fig. 5C**). In response to IL-12+IL-18, recruitment of RNA Pol II to the *Ifng* promoter, first exon and first intron was significantly impaired in the absence of CNS-22, suggesting that the ability of CNS-22 to enhance *Ifng* transcription is linked to its ability to up-regulate HAT activity. In contrast, while CNS-22–deficient Th1 cells showed a modest decrement in Pol II recruitment to the promoter and first exon of *Ifng* in response to TCR signaling, this did not achieve statistical significance (**Fig. 5C**). Together, these results suggest that *trans*-factor recruitment to CNS-22 downstream of IL-12+IL-18 signaling induced increased acetylation that was largely independent of enhanced p300 recruitment. These results further suggest that inducible acetylation at distal enhancers that regulate *Ifng* transcription is directly linked to their ability to modulate recruitment of RNA Pol II to the *Ifng* gene in response to external stimuli.

## Discussion

CNS-22 was previously identified as an important *cis*-regulatory element hypothesized to have a central role in epigenetic remodeling of the *Ifng* locus during lineage-specific Th1, Tc1 and NK cell differentiation [13], [18]. In the current study, CNS-22–deficient mice were generated to enable study of the consequences of deletion of this element for epigenetic remodeling of the endogenous *Ifng* locus. Here we identify CNS-22 as critical element for early remodeling of the *Ifng* locus in naïve T cells and establish its importance as an enhancer for optimal *Ifng* transcription in Th1, Tc1 and NK cells. However, we find that the epigenetic consequences of the deletion of CNS-22 during lineage specification are limited to circumscribed effects on critical upstream regulatory elements surrounding CNS-22, such that remodeling of regulatory elements downstream of the *Ifng* gene occurs largely independently of CNS-22 in Th1 or Tc1 cells. Unexpectedly, fine mapping of the epigenetic consequences of CNS-22 deletion led to the finding that distal *cis*-acting elements activate inducible gene transcription through hyperacetylation of nucleosomes that flank *trans*-factor binding core elements of enhancers. This supports a model of eukaryotic enhancer function wherein the activity and/or composition of HAT complexes loaded onto core enhancer elements during lineage-specific differentiation are rapidly modulated in concert with activation-induced *trans*-factor recruitment to effect increased gene transcription.

Speculation that CNS-22 is a central node for permissive remodeling of the extended *Ifng* locus arose from our previous studies wherein deletion of CNS-22 from an *Ifng-Thy1.1* BAC reporter transgene caused nearly complete ablation of reporter expression in Th1, Tc1 and NK cells [13], [18]. In the present study, deletion of CNS-22 from the endogenous *Ifng* locus led to a substantial defect in induction of *Ifng*, albeit less pronounced than the same deletion from the BAC transgene. Although several factors could account for the disparities observed, the exclusion from our *Ifng* BAC transgene of boundary elements located −70 kb upstream and the new potential boundary element identified herein located 342 kb downstream of the *Ifng* gene leads us to speculate that absence of these architectural elements might have compromised the efficiency of approximation of the CNS-22 enhancer to the core promoter mediated by a CTCF-cohesin–dependent mechanism. This might well have resulted in an exaggerated loss of *Ifng* transcription upon deletion of CNS-22 from the BAC transgene [17], [18]. Also missing from the BAC transgene were hypersensitive sites identified herein located +105, +151 and +159 kb downstream of the transcriptional state site of the *Ifng* gene. All three of these elements are likely to be functional, as the first two co-recruit CTCF and Smc3 while the site at +159 recruits STAT4. Any or all of these could contribute to the exaggerated expression defect observed on deletion of CNS-22 in from the BAC-transgene. Finally, it should be noted that *Tmevp3*, a gene that encodes a long intergenic non-coding RNA (lincRNA) that maps to ∼+60 kb to +120 kb downstream of the *Ifng* gene was excluded in the *Ifng-Thy1.1* BAC transgene [38]. Although two recent studies document that the lincRNA encoded by *Tmevp3* acts in *trans* to recruit H3K4 methyltransferases to the *Ifng* locus, it is possible, although we think it unlikely, that exclusion of *Tmevp3* in the *Ifng-Thy1.1* transgene might have compromised transcriptional activation upon deletion of CNS-22 in this context [39], [40]. Suffice it to say that further studies will be required to precisely define the basis for the observed expression disparities and should be informative.

Importantly, deletion of CNS-22 from endogenous *Ifng* alleles enabled analyses of developmental stage-specific effects on chromatin organization not previously possible using the BAC transgenic model. Thus, while CNS-22^−/−^ naïve CD4^+^ T cells demonstrated compromised development of several HS sites present in WT cells, including those at CNSs +17–19 and +46, as well as H3K4 methylation of CNS-34, Th1 differentiation largely overrode these defects. This indicates that developmentally regulated HS sites that dictate low-level *Ifng* expression competence characteristic of naïve CD4^+^ T cells are dependent upon the presence CNS-22, exposing an important role for CNS-22 in early remodeling of the locus. However, differentiation that confers high-level *Ifng* transcriptional activity in Th1 and Tc1 cells proceeds such that a number of key HS sites, primarily those downstream of the *Ifng* gene, can be remodeled independently of CNS-22. Thus, although CNS-22 remains an important element for differentiation-dependent remodeling of the extended *Ifng* locus, its influence is not global, rather it is more local, consistent with the previously proposed organization of the *Ifng* locus into upstream and downstream regulatory domains coordinated by recruitment of CTCF boundary elements to the intronic CTCF element within the *Ifng* gene [19], [20].

CNS-22 recruits at least four key transcription factors to activate *Ifng* transcription: STAT4 [29], T-bet [18], Runx3 [28] and RelA [29]. We predict that defects in *Ifng* induction in CNS-22^−/−^ T and NK cells stems from deletion of the STAT4 binding site in CNS-22. STAT4 is activated downstream of the IL-12 receptor and plays an essential role in IL-12 dependent polarization of naïve CD4^+^ T cells to IFN-γ-competent Th1 cells [41], [42]. IL-12+IL-18 dependent induction of *Ifng* is also absolutely dependent on activation of STAT4 [43]. In addition to the impaired IL-12+IL-18 driven induction of *Ifng* in CNS-22^−/−^ Th1 cells, we also document that acquisition of competency of the *Ifng* locus is considerably delayed in the absence of CNS-22. At least three STAT4 recruiting modules, CNSs −22, +40 and +46 are accessible in naïve CD4^+^ T cells [25]. During the course of Th1 differentiation, additional STAT4 recruiting modules at the *Ifng* promoter, CNSs −34, +30 and +54 also become accessible [29]. It is likely that sustained IL-12 signaling combined with CNS+40- and +46-dependent remodeling of other STAT4 binding modules compensate for the absence of CNS-22. Nonetheless, subsequent deficiency in IL-12+IL-18 dependent induction of *Ifng* highlights the importance of the STAT4 binding site within CNS-22. Further studies to mutate and evaluate individual transcription factor binding sites within CNS-22 would provide new insights into the roles of individual *trans* factor binding sites within CNS-22.

An important finding of this study is the identification of a link between activation-dependent recruitment of HAT activity and enhancer function. Several previous studies have linked acetylation of histones to differentiation-dependent activation of gene transcription, including the recently discovered association between H3K27ac and enhancer actions [35]. In addition, at least two bromodomain-containing HATs — p300 [36], [37] and CBP [36] — have been demonstrated to regulate lineage-specific activation of enhancers. Here, we establish for the first time a clear relationship between enhancer actions and acute increases in HAT activity by demonstrating that CNS-22 not only dictates activation-induced acquisition of H4K12ac marks in its immediate vicinity, but also regulates acquisition of H4K12ac marks at multiple distal sites. Together, these results suggest that CNSs across the *Ifng* locus interact with each other and coordinate activation-driven recruitment of HATs via long-range interactions. Moreover, by evaluating IL-12+IL-18 dependent RNA Pol II recruitment in CNS-22^−/−^ Th1 cells, we demonstrate that impaired hyperacetylation compromises the ability of CNS-22 deficient Th1 cells to recruit RNA Pol II to the proximal *Ifng* promoter in response to IL-12+IL-18. Notably, the marked CNS-22–dependent increases in histone acetylation that followed IL-12+IL-18 signaling, and to a lesser extent TCR-mediated signaling, proceeded without substantive changes in the levels of p300 binding at these elements. This is consistent with a role for acute *trans*-factor-dependent recruitment of other HATs and/or activation of pre-loaded p300 HAT complexes to increase histone acetylation and Pol II loading on the promoter, and will require further study.

In summary, studies herein have delineated a role for CNS-22 in regulating epigenetic changes that control chromatin remodeling and transcriptional competence of the *Ifng* locus prior to, during and subsequent to lineage specification. CNS-22, while essential for locus remodeling in naïve T cells and optimal *Ifng* transcription in mature effector T cells, has a more restricted function in developmentally driven remodeling of the *Ifng* locus than previously thought, with its principal effects being exerted on regulatory elements bounded by the upstream and intronic CTCF elements thought to be important in approximating these upstream elements to the proximal promoter. Further, analysis of T cells from CNS-22^−/−^ mice has uncovered a previously unappreciated role of HATs in modulating actions of eukaryotic enhancers for induction of high-level transcription that is characteristic of cytokine genes. Our findings suggest that recruitment and activation of HATs at CNS-22 and other distal enhancers in the *Ifng* locus occurs in two stages: the first, an initial wave to induce basal acetylation of enhancer-associated histones that confers receptiveness to a second, subsequent wave of *trans*-factor–induced hyperacetylation that is linked to high-level *Ifng* transcription. Although further studies will be necessary, these findings support a model in which a conserved regulatory module, such as CNS-22, contains a core enhancer that includes sites for *trans*-factor binding contingent upon differentiation-dependent nucleosomal clearing or remodeling, whereas the flanking nucleosomes constitute part of an extended enhanceosome that is less evolutionarily constrained. While whole genome analyses of the epigenome have rapidly advanced our understanding of differentiation-dependent alterations in chromatin remodeling and identified important epigenetic markers that have enabled identification of potential enhancers, detailed locus-specific analyses of the type exemplified herein for the *Ifng* locus will be important to complement our understanding of *cis*-element function moving forward.

## Materials and Methods

### Ethics statement

All animal studies were conducted in accordance with guidelines and oversight of the institutional animal use and care committee of University of Alabama at Birmingham.

### Mice, antibodies and reagents

C57BL/6 and OT-II TCR transgenic mice were purchased from Jackson Laboratory and/or bred at the University of Alabama at Birmingham. Generation of CNS-22^−/−^ mice is described in the supplement. Primers used for PCR-based screening have been previously described [18]. Antibodies against H3K4me1,2,3 (04-791), H3K4me2 (17-677), H3K4me3 (17-678), Pan H4 (08-858), H4 acetyl (06-598), H3K9ac (17-658) and H4K12ac (07-595) and RNA polymerase II (05-952) were purchased from Millipore (Billlerica, MA). Antibodies against p300 (sc-584, sc-585) were purchased from Santa Cruz Biotechnology (Santa Cruz, CA). All primers and probes were synthesized by IDT (Coralville, IA).

### Naïve cell sorting and generation of Th1, Th2, Th17 and Tc1 cells

FSC^lo^, CD62L^hi^, CD44^lo^ cells were purified from CD4^+^ T cells isolated by positive selection from spleen and pooled lymph nodes. For generation of Th cells, CD4^+^ T cells were isolated by positive selection from spleen and pooled lymph nodes. Differentiation of Th1, Th2, Th17 and Tc1 cells was performed as previously described [25], [29], [44], [45]. Positive selection was carried out using CD4-DYNAL beads (Invitrogen) or anti-PE microbeads (Miltenyi). Reactivation of cells for ChIP has been previously described [29]. Briefly, Th1, Tc1 or NK cells were activated with 10 ng/ml rIL-12 and 25 ng/ml rIL-18. For TCR restimulation, anti-CD3 antibody was diluted to 10 µg/ml in phosphate buffered saline (PBS, 150 mM NaCl, 0.02M Phosphate) and coated overnight at 4°C. The following day the plates were was washed twice with PBS and the media was supplemented with 5 µg/ml of anti-CD28 antibody.

### Intracellular cytokine staining

Cells were reactivated for 4 hours in the presence of GolgiStop (BD Biosciences; San Jose, CA) as per manufacturer's recommendations and stained with fluorescent-labeled antibodies against CD4, CD8, IL-4 and IFN-γ using the Cytofix/Cytperm kit (BD Biosciences). Dead cells were excluded by staining with LIVE/DEAD fixable stain kits (Invitrogen; Carlsbad, CA). Samples were acquired on an LSRII flow cytometer and analyzed using FlowJo software (Treestar Inc.; Ashland, OR).

### Chromatin immunoprecipitation, DNase-chip and ChIP-chip

Protocols employed for ChIP, ChIP-chip DNase-chip have been previously described [25], [29]. Primer sequences that were not previously published [25] are available upon request. We employed a slightly modified ChIP protocol for p300 ChIP. Cells were dounced in 25 mM Hepes (pH 7.8), 1.5 mM MgCl2, 10 mM KCl, 0.1% NP-40, 1× complete protease inhibitor cocktail (Roche). Nuclei were isolated, resuspended in 0.1× SDS lysis buffer (Millipore) diluted in 1× ChIP dilution buffer (Millipore). Samples were sonicated in and subject to ChIP as previously described using ChIP assay kit (Millipore). For ChIP-chip, samples and inputs were amplified using WGA2 kit (Sigma), labeled and hybridized to custom-designed microarrays (Roche-Nimblegen). A previously described algorithm, ACME was employed for peak calling and identifying enriched regions in the ChIP-chip datasets [46].

### Transcript analyses

RNA was isolated using RNeasy kit (Qiagen), subject to DNA*free* treatment (Applied Biosystems) to remove any contaminating DNA. RNA was then reverse-transcribed using Superscript III cDNA synthesis kit (Invitrogen) and transcript levels were normalized against housekeeping gene β2-microglobulin.

## Supporting Information

Figure S1 Conserved elements in the *Ifng* locus and generation of *Ifng*-CNS-22^−/−^ mice. (**A**) Syntenic regions of human and murine *Ifng* gene loci are shown aligned using the VISTA browser. (**B**) A galK based selection strategy was used to incorporate a loxP site upstream of CNS-22 into BAC clone 348O11RP­24 [18]. A frt-flanked neomycin resistance gene flanked by a single loxP site was recombineered at the 3′ end of CNS-22. Construct integrity was verified by restriction enzyme analyses and sequencing and then electroporated into Bruce4 ES cells. Targeted ES clones identified by southern blotting were expanded, injected into albino B6 blastocysts and transferred into pseudo-pregnant females to obtain chimeric mice. (**C**) DNase I hypersensitivity tracks from Fig. 1A have been zoomed in to highlight the fact that only the conserved core sequence within CNS-22 was deleted to generate CNS-22^−/−^ mice.
(TIF)

Figure S2 Concentration-dependent effects of IL-12 priming on *Ifng* transcription deficit in CNS-22–deficient T cells. (**A**) Naïve CD4^+^ T cells isolated from OT-II transgenic WT and CNS-22^−/−^ mice were differentiated with ova-peptide and CD4-depleted irradiated feeder cells derived from *Il12a* ^−/−^ mice supplemented with addition of 20 ng/ml, 2 ng/ml or 0 ng/ml IL-12. Cells were recovered on day 5 and reactivated with IL-12+IL-18 or anti-CD3/CD28 as described in Methods. Frequencies of viable, IFN-γ^+^ T cells were determined by flow cytometric analysis. Titration of the doses of IL-12 and IL-18 used during restimulation did not significantly alter the relative expression differences observed (data not shown). Numbers indicate percentages of IFN-γ positive cells within viable CD4^+^ T cell gates. Data are representative of at least two independent experiments. (**B**) CD4^+^ T cells isolated from OT-II transgenic WT and CNS-22^−/−^ mice were differentiated as in **A** using 2 ng/ml IL-12, then restimulated with IL-12+IL-18 for 4 hours. RNA was isolated, reverse transcribed and levels of *Tbx21* and *Runx3* were assessed by real-time PCR. Transcript levels were normalized against levels of β2 microglobulin.
(TIF)

Figure S3 Impaired type I immune response in CNS-22-deficient mice. To assess the effects of CNS-22 deficiency on IFN-γ expression by T cells and NK cells in vivo, a *Listeria monocytogenes* (Lm) infection model was used, as described [49]. WT and CNS-22^−/−^ OT-II TCR transgenic mice were inoculated i.v. with 1×10^6^ Lm that express OVA peptide (Lm-OVA). Eight days following inoculation, spleens were recovered from infected WT and infected CNS-22^−/−^ mice, or uninfected WT controls, and stimulated ex vivo for 4 h with anti-CD3 or OVAp, then assessed for intracellular IFN-γ expression as described in Methods (**A**, **B**). Representative flow cytometric plots of splenocytes stimulated with anti-CD3 are shown in (**A**), with numbers indicating the frequencies of IFN-γ^+^ CD4^+^ T cells. (**B**) Composite data of IFN-γ^+^CD4^+^ T cells recovered splenocytes stimulated with anti-CD3 or OVAp. * p<0.01, # p<0.05; stimulated CNS-22^−/−^ relative to WT. (**C**) Eight days following inoculation, WT and CNS-22^−/−^ mice were re-challenged with Lm-OVA i.v., 3 h after which they received brefeldin A i.p.. One hour later spleens were recovered and processed for assessment of intracellular IFN-γ expression on lymphoid-cell gated subpopulations gated for co-expression of CD3^+^CD4^+^CD8α^−^ (CD4^+^ T cells), CD3^+^CD4^−^CD8α^+^ (CD8^+^) or CD3^−^NK1.1^+^ (NK cells). Shown are the geometric mean fluorescence intensity (MFI) of intracellular IFN-γ expression by the indicated. * p<0.01, # p<0.05, WT versus CNS-22^−/−^. Data for in vivo studies are representative of at least two independent experiments.
(TIF)

Figure S4 Differential acetylation identifies lineage-specific acquisition of transcriptional competence at multiple T cell cytokine gene loci. CD4^+^ T cells isolated from OT-II^+^ TCR transgenic mice were cultured under Th2 and Th17 differentiation conditions for 5 days. Levels of H4K12ac at the *Il4-Il13-Il5* and *Il17a-Il17f* gene loci were assessed by ChIP-chip. These data are shown aligned against averaged DNase I tracks of Th2 (**A**) and Th17 cells (**B**). ACME peak calling thresholds were set to a confidence limit of 95% for all datasets as described in Fig. 3. Data are representative of at least two independent experiments.
(TIF)

Figure S5 Induction of *Ifng* transcription is associated with CNS-22-dependent acetylation of flanking nucleosomes. Th1 cells derived from WT and CNS-22^−/−^ mice were subject to ChIP using an antibody that recognizes acetylated histone H4. Relative H4 acetylation levels were calculated by comparisons with no antibody controls and are represented as a fraction of the H4 acetylation observed at 16Srp promoter, which was assigned a value of 1. Cells were unactivated (**A**) or were activated for 3 h with either anti-CD3+anti-CD28 antibodies (**B**) or IL-12+IL-18 (**C**). Data are representative of at least two independent experiments. Statistical analyses were carried out on means and standard errors from three independent experiments * p<0.01, # p<0.05, stimulated WT versus CNS-22^−/−^.
(TIF)

Figure S6 p300 is recruited to multiple enhancers that regulate *Ifng* transcription. p300 recruitment across the extended *Ifng* locus was mapped using ChIP-chip in WT Th1 and Tc1 cells that were either left unstimulated or activated with IL-12 and IL-18 for 1.5 h. Peak-calling was carried out as described in Fig. 3. Data are representative of at least two independent experiments.
(TIF)
