# Supplementary figures and images for: Deletion of a Conserved cis-Element in the Ifng Locus Highlights the Role of Acute Histone Acetylation in Modulating Inducible Gene Transcription
Source: PLoS Genet. 2014 Jan 9;10(1):e1003969. doi: 10.1371/journal.pgen.1003969 (PMC3886902; doi:10.1371/journal.pgen.1003969)

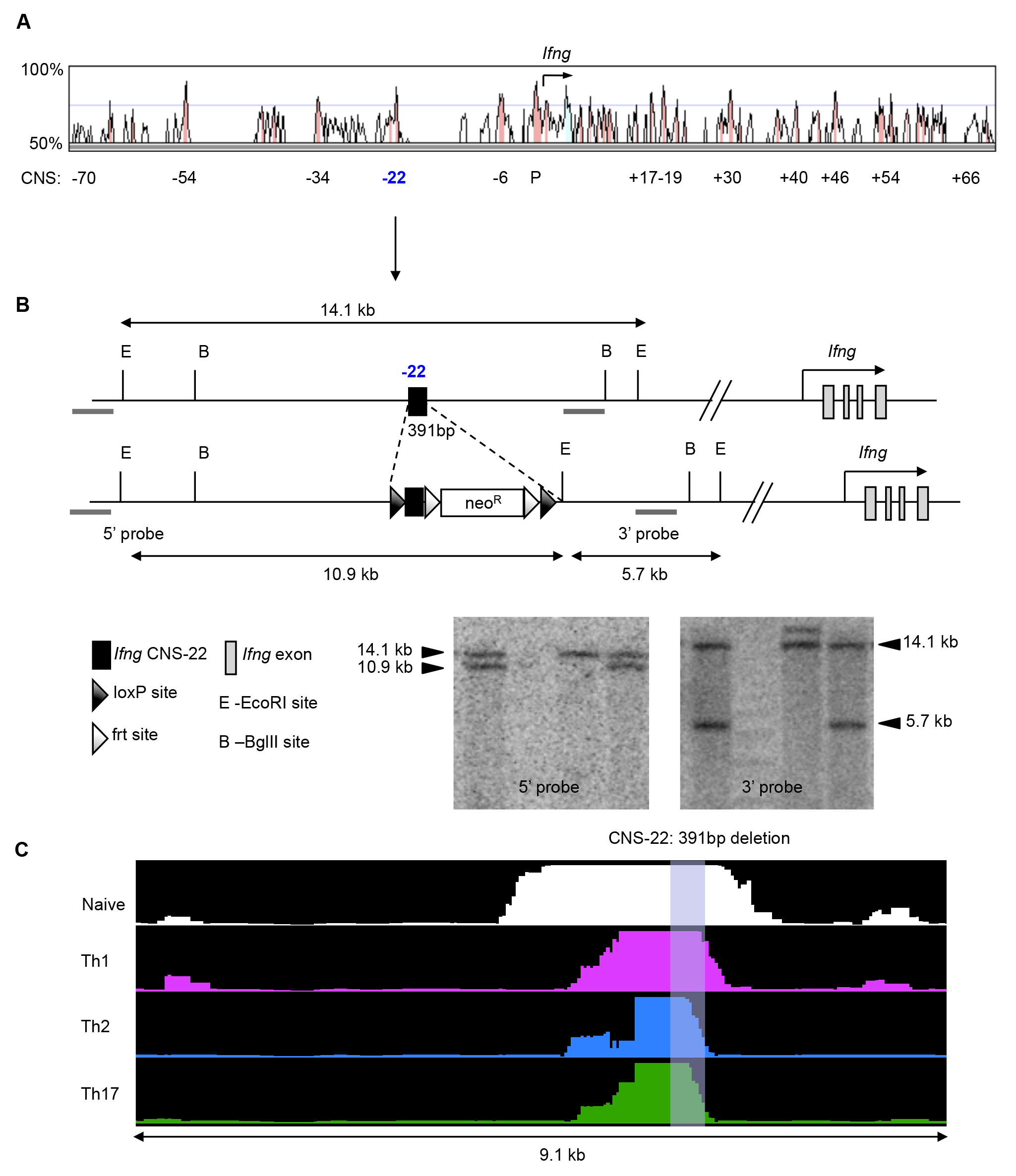

Supplement: Figure S1 — Conserved elements in the Ifng locus and generation of Ifng-CNS-22−/− mice. (A) Syntenic regions of human and murine Ifng gene loci are shown aligned using the VISTA browser. (B) A galK based selection strategy was used to incorporate a loxP site upstream of CNS-22 into BAC clone 348O11RP­24 [18]. A frt-flanked neomycin resistance gene flanked by a single loxP site was recombineered at the 3′ end of CNS-22. Construct integrity was verified by restriction enzyme analyses and sequencing and then electroporated into Bruce4 ES cells. Targeted ES clones identified by southern blotting were expanded, injected into albino B6 blastocysts and transferred into pseudo-pregnant females to obtain chimeric mice. (C) DNase I hypersensitivity tracks from Fig. 1A have been zoomed in to highlight the fact that only the conserved core sequence within CNS-22 was deleted to generate CNS-22−/− mice. (TIF) [file pgen.1003969.s001.tif]

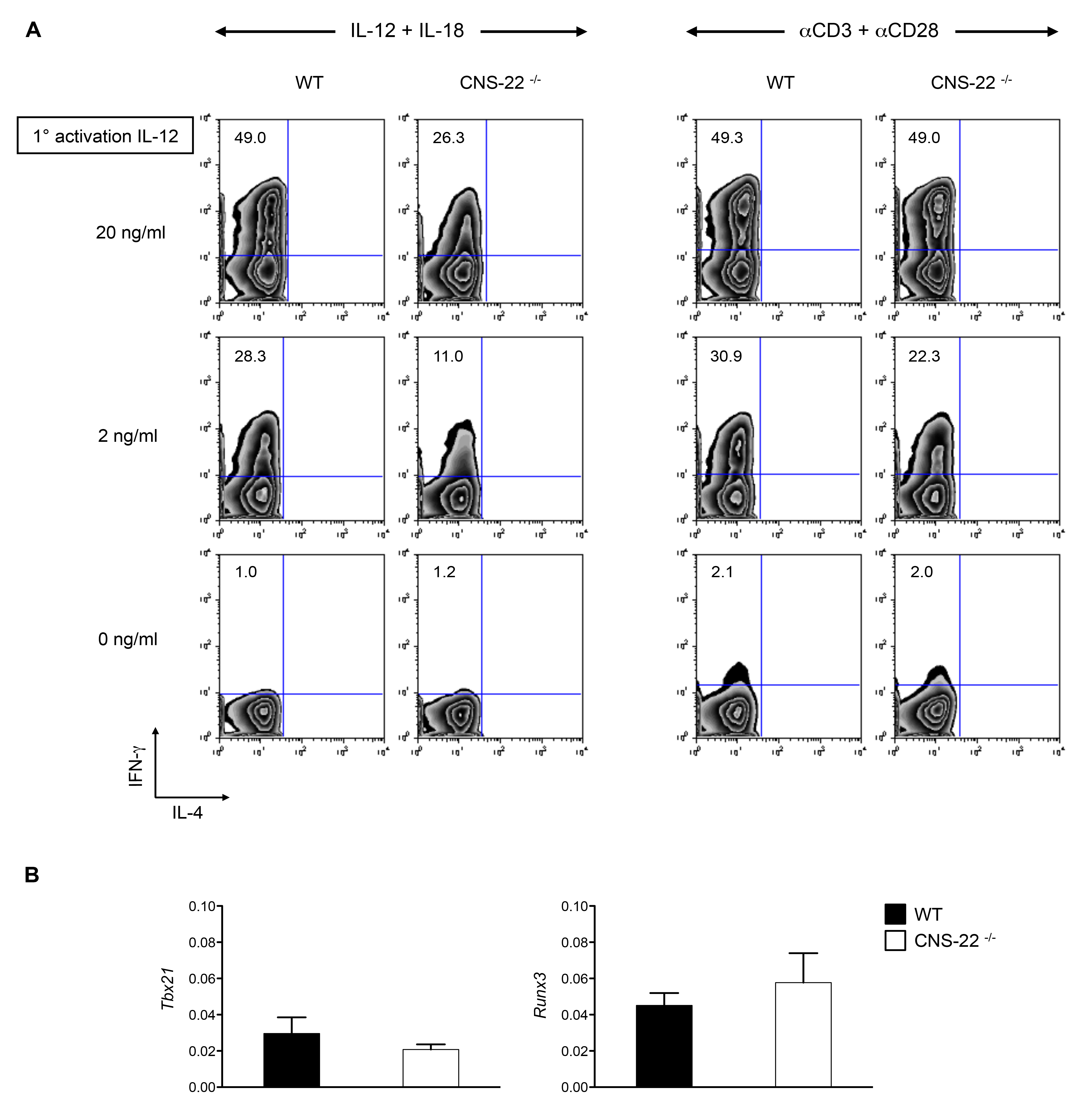

Supplement: Figure S2 — Concentration-dependent effects of IL-12 priming on Ifng transcription deficit in CNS-22–deficient T cells. (A) Naïve CD4+ T cells isolated from OT-II transgenic WT and CNS-22−/− mice were differentiated with ova-peptide and CD4-depleted irradiated feeder cells derived from Il12a −/− mice supplemented with addition of 20 ng/ml, 2 ng/ml or 0 ng/ml IL-12. Cells were recovered on day 5 and reactivated with IL-12+IL-18 or anti-CD3/CD28 as described in Methods. Frequencies of viable, IFN-γ+ T cells were determined by flow cytometric analysis. Titration of the doses of IL-12 and IL-18 used during restimulation did not significantly alter the relative expression differences observed (data not shown). Numbers indicate percentages of IFN-γ positive cells within viable CD4+ T cell gates. Data are representative of at least two independent experiments. (B) CD4+ T cells isolated from OT-II transgenic WT and CNS-22−/− mice were differentiated as in A using 2 ng/ml IL-12, then restimulated with IL-12+IL-18 for 4 hours. RNA was isolated, reverse transcribed and levels of Tbx21 and Runx3 were assessed by real-time PCR. Transcript levels were normalized against levels of β2 microglobulin. (TIF) [file pgen.1003969.s002.tif]

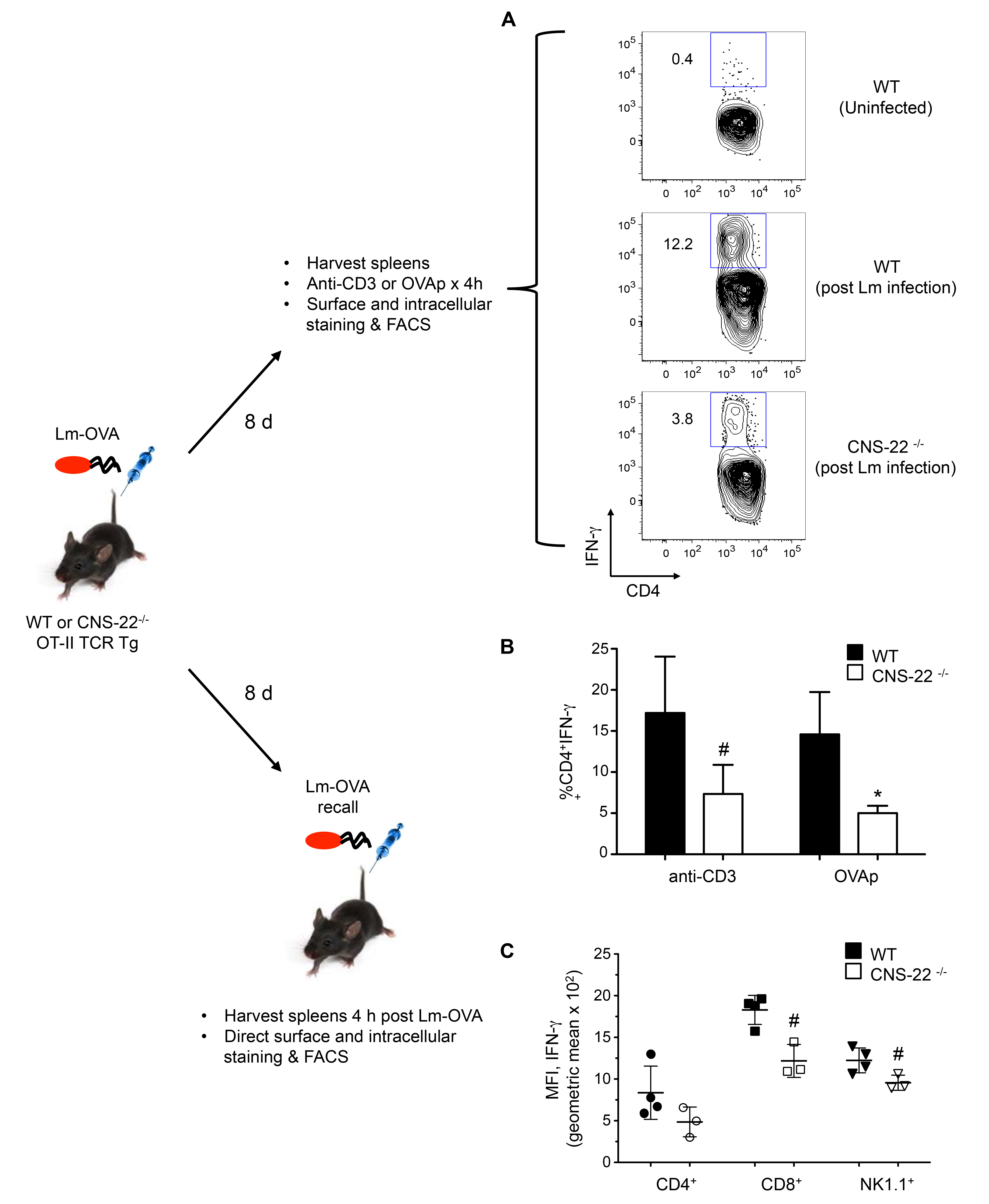

Supplement: Figure S3 — Impaired type I immune response in CNS-22-deficient mice. To assess the effects of CNS-22 deficiency on IFN-γ expression by T cells and NK cells in vivo, a Listeria monocytogenes (Lm) infection model was used, as described [49]. WT and CNS-22−/− OT-II TCR transgenic mice were inoculated i.v. with 1×10^6^ Lm that express OVA peptide (Lm-OVA). Eight days following inoculation, spleens were recovered from infected WT and infected CNS-22−/− mice, or uninfected WT controls, and stimulated ex vivo for 4 h with anti-CD3 or OVAp, then assessed for intracellular IFN-γ expression as described in Methods (A, B). Representative flow cytometric plots of splenocytes stimulated with anti-CD3 are shown in (A), with numbers indicating the frequencies of IFN-γ+ CD4+ T cells. (B) Composite data of IFN-γ+CD4+ T cells recovered splenocytes stimulated with anti-CD3 or OVAp. * p<0.01, # p<0.05; stimulated CNS-22−/− relative to WT. (C) Eight days following inoculation, WT and CNS-22−/− mice were re-challenged with Lm-OVA i.v., 3 h after which they received brefeldin A i.p.. One hour later spleens were recovered and processed for assessment of intracellular IFN-γ expression on lymphoid-cell gated subpopulations gated for co-expression of CD3+CD4+CD8α− (CD4+ T cells), CD3+CD4−CD8α+ (CD8+) or CD3−NK1.1+ (NK cells). Shown are the geometric mean fluorescence intensity (MFI) of intracellular IFN-γ expression by the indicated. * p<0.01, # p<0.05, WT versus CNS-22−/−. Data for in vivo studies are representative of at least two independent experiments. (TIF) [file pgen.1003969.s003.tif]

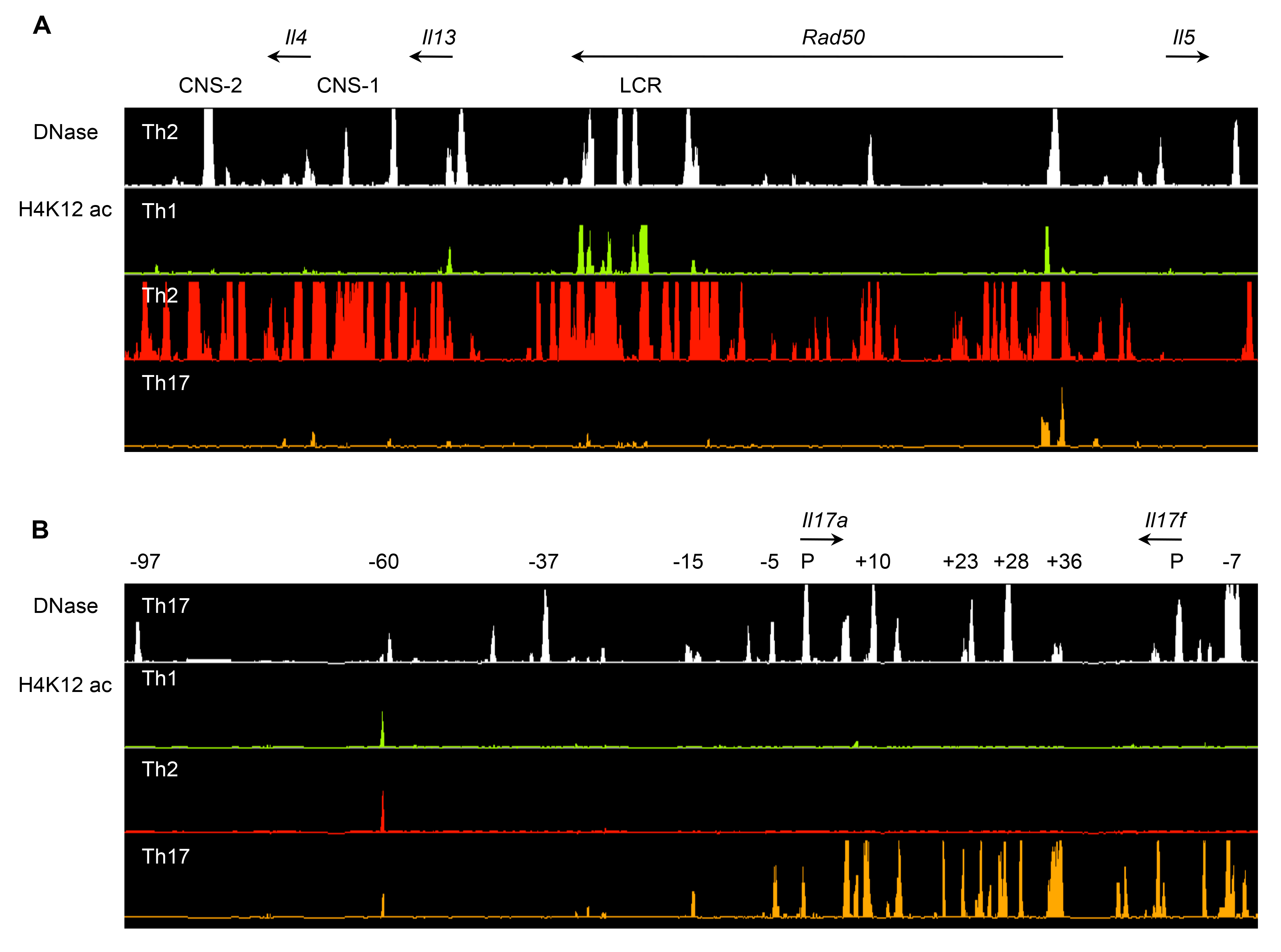

Supplement: Figure S4 — Differential acetylation identifies lineage-specific acquisition of transcriptional competence at multiple T cell cytokine gene loci. CD4+ T cells isolated from OT-II+ TCR transgenic mice were cultured under Th2 and Th17 differentiation conditions for 5 days. Levels of H4K12ac at the Il4-Il13-Il5 and Il17a-Il17f gene loci were assessed by ChIP-chip. These data are shown aligned against averaged DNase I tracks of Th2 (A) and Th17 cells (B). ACME peak calling thresholds were set to a confidence limit of 95% for all datasets as described in Fig. 3. Data are representative of at least two independent experiments. (TIF) [file pgen.1003969.s004.tif]

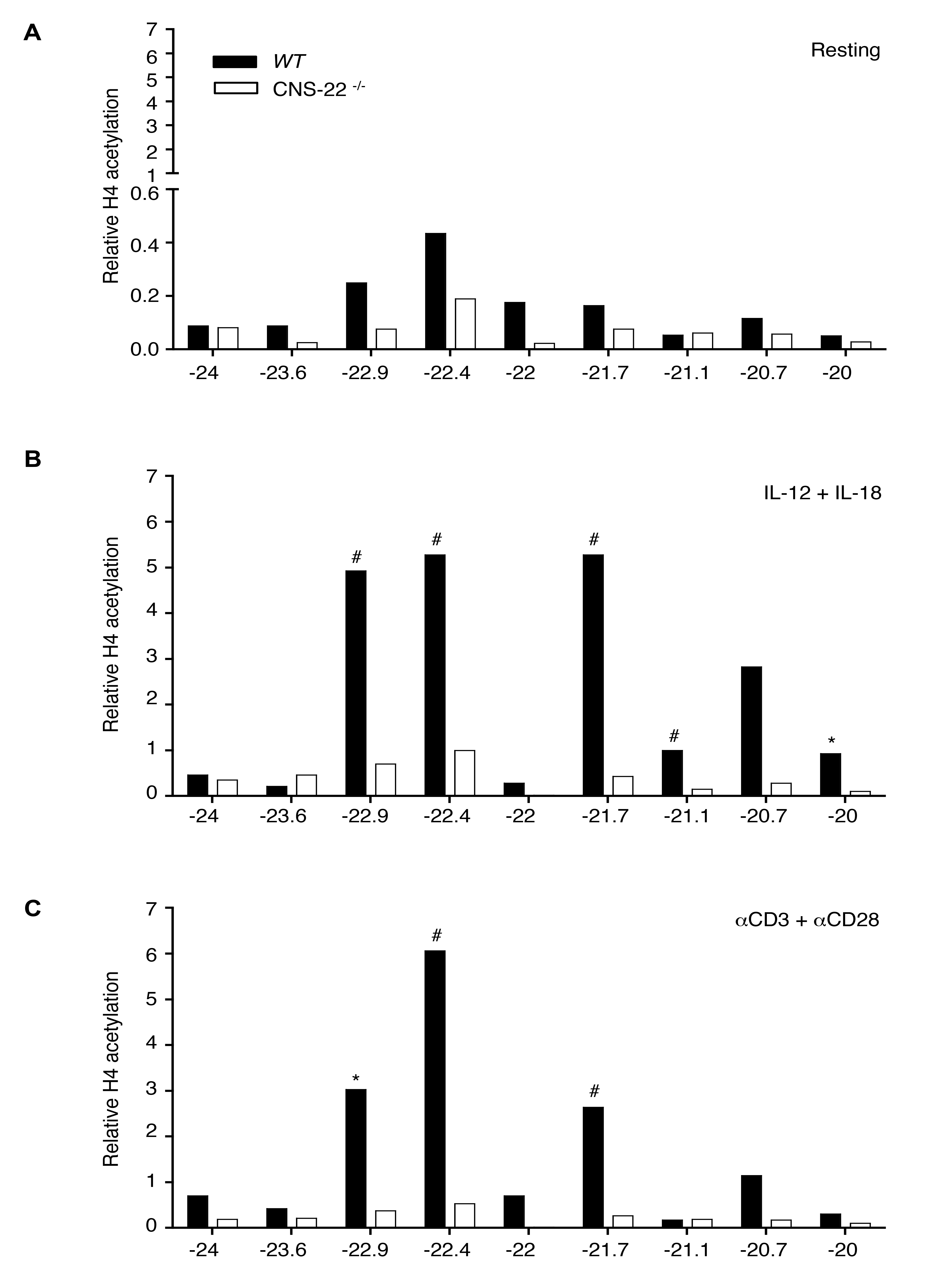

Supplement: Figure S5 — Induction of Ifng transcription is associated with CNS-22-dependent acetylation of flanking nucleosomes. Th1 cells derived from WT and CNS-22−/− mice were subject to ChIP using an antibody that recognizes acetylated histone H4. Relative H4 acetylation levels were calculated by comparisons with no antibody controls and are represented as a fraction of the H4 acetylation observed at 16Srp promoter, which was assigned a value of 1. Cells were unactivated (A) or were activated for 3 h with either anti-CD3+anti-CD28 antibodies (B) or IL-12+IL-18 (C). Data are representative of at least two independent experiments. Statistical analyses were carried out on means and standard errors from three independent experiments * p<0.01, # p<0.05, stimulated WT versus CNS-22−/−. (TIF) [file pgen.1003969.s005.tif]

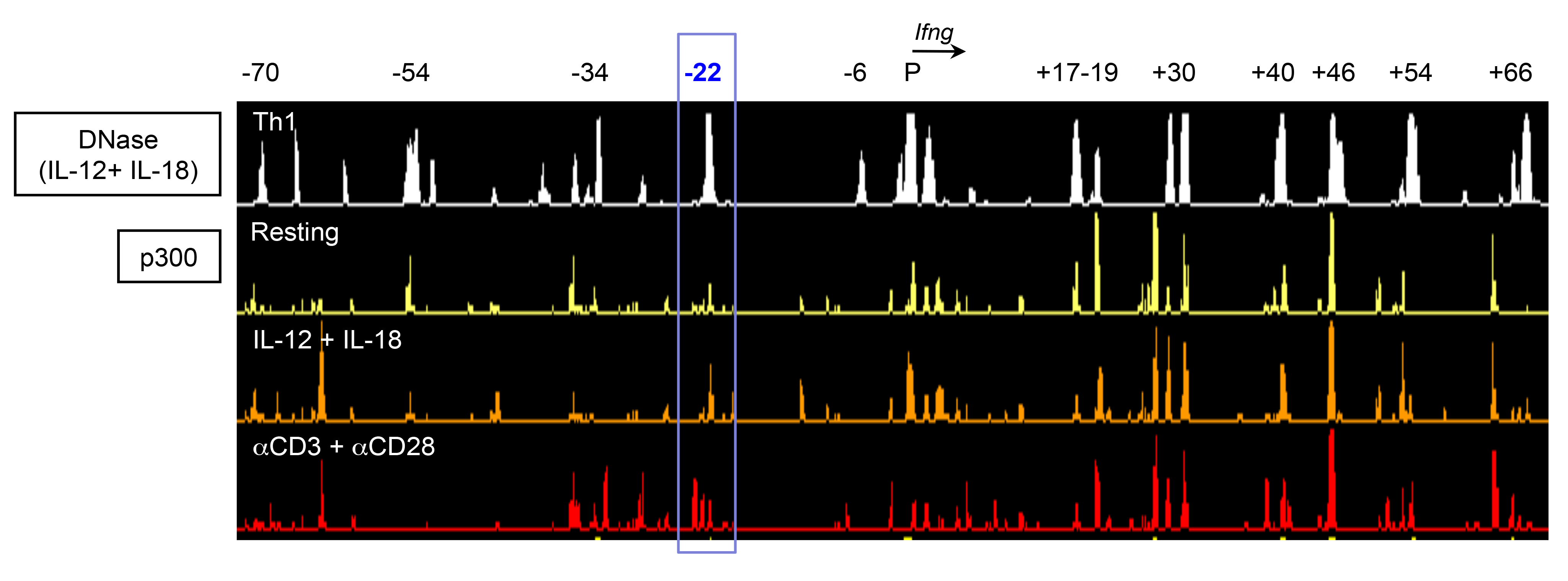

Supplement: Figure S6 — p300 is recruited to multiple enhancers that regulate Ifng transcription. p300 recruitment across the extended Ifng locus was mapped using ChIP-chip in WT Th1 and Tc1 cells that were either left unstimulated or activated with IL-12 and IL-18 for 1.5 h. Peak-calling was carried out as described in Fig. 3. Data are representative of at least two independent experiments. (TIF) [file pgen.1003969.s006.tif]
